# Epigastric Heteropagus Twin

**DOI:** 10.21699/jns.v6i2.491

**Published:** 2017-04-15

**Authors:** Prince Raj, Hirendra Birua

**Affiliations:** Department of Pediatric Surgery, Rajendra Institute of Medical Sciences, Ranchi

A 1-day-old, full-term 3.9kg male baby, was born with difficult vaginal delivery leading to third degree vaginal tear to a 26-year-old mother. On examination, the parasite was attached to the epigastrium of the autosite and it possessed two rudimentary upper limbs, two well-developed lower limbs, a pelvis, and a well-developed scrotum and penis, which produced urine discharge and an imperforate anus (Fig.1A). X-ray showed developed bones in the lower limbs of the parasite, whereas there was fracture of the right humerus of the host baby due to difficult delivery. The contrast enhanced computed tomography (CECT) showed the parasite having a single pelvic kidney and few bowel loops (Fig.1B). Echocardiography of the host showed a patent ductus arteriosus. Surgery to separate the twins was performed on 28^th^ day of life. The parasitic twin was connected to the sternum of the autosite by a tract of cartilage. Furthermore, parasite was sharing the liver with the autosite. The extrahepatic bile duct system of the parasite was separated after ligating it. The main vascular pedicle of the parasite originated from the falciform ligament of the autosite (Fig.1C). The pelvis of the parasite contained single functioning kidney, a urinary bladder, and a small intestine but lacked a large intestine and anus (Fig.1D). The small intestine of the parasite displayed proximal atresia and was connected distally to the bladder like structure. There was 0.5x0.5cm perforation in parasitic gut probably due to closed gut obstruction. The weight of the separated parasite was 1200g. 

Epigastric heteropagus twins (EHT) are rare type of monozygotic monochorionic asymmetrical conjoined twins. Embryological basis of EHT has been debated between the incomplete fission versus the fusion theory. It is generally considered to be a product of error in blastogenesis by incomplete fission of a single zygote post 14 days of fertilization.[1] Whereas proponents of fusion theory consider it due to fusion of two embryos.[2,3] 

EHTs are generally common in males accounting for almost 3/4^th^ of all the reported cases.[4] Ours was also a male baby which goes in accordance with the published statistic. In the current era of mandatory antenatal check ups and sonography, almost all cases are diagnosed in-utero during the anomaly scan. Thus proper parental counseling is done as regards to the condition and its outcome. Planning is also made regarding the mode of delivery based on the size of the parasitic twin. But in developing nation like ours where mostly the scan is done by the treating obstetrician or is not done at all, this major rare anomaly is still missed. This happened in our case too, where in spite of two antenatal scans done by the treating obstetrician the parasite twin was missed. This resulted in complicated child birth with fracture of right humerus of autosite and third degree vaginal tear in mother (though she was fourth gravida) requiring repair.

## Footnotes

**Source of Support:** None

**Conflict of Interest:** None

## Figures and Tables

**Figure 1:**
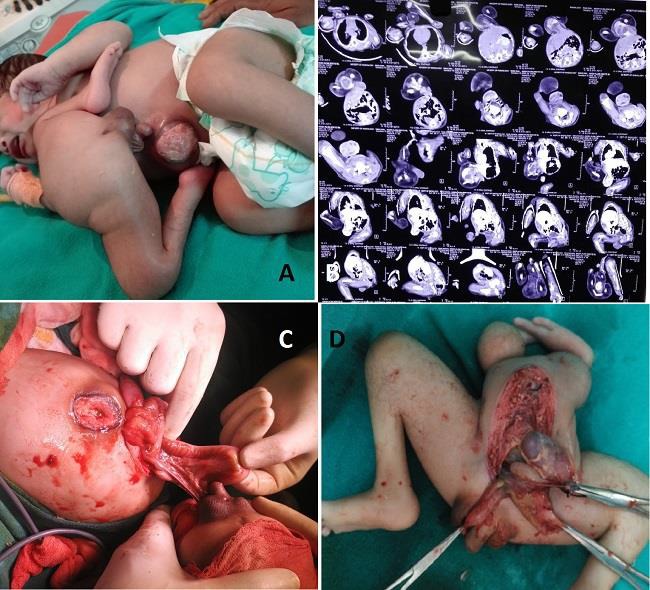
A) Epigastric heteropagus with well developed lower limb, phallus, scrotum and anal dimple in the parasitic twin with omphalocele in the autosite. B) CECT. C) Intraoperative details showing 20 cm of bowel present closed at both ends. D) Resected specimen showing bladder, intestine connected to the bladder and solitary kidney with rudimentary upper limb and well developed lower limb.
